# Definitive radiotherapy alone over 60 Gy for patients unfit for combined treatment to stage II-III non-small cell lung cancer: retrospective analysis

**DOI:** 10.1186/s13014-015-0560-z

**Published:** 2015-12-03

**Authors:** Ji Hyeon Joo, Si Yeol Song, Su Ssan Kim, Yuri Jeong, Seong-Yun Jeong, Wonsik Choi, Eun Kyung Choi

**Affiliations:** Department of Radiation Oncology, Asan Medical Center, University of Ulsan College of Medicine, 88, Olympic-ro 43-gil, Songpa-Gu, 138-736 Seoul Korea; Institute for Innovative Cancer Research, Asan Medical Center, University of Ulsan College of Medicine, Seoul, Korea; Department of Radiation Oncology, Gangeung Asan Hospital, University of Ulsan College of Medicine, Gangneung, Korea

**Keywords:** NSCLC, Radiotherapy alone, Definitive, Old age, Local control, Tumor response

## Abstract

**Background:**

Elderly patients with non-small cell lung cancer (NSCLC) are frequently treated with radiation therapy (RT) alone, due to poor performance status or underlying disease. We investigated the effectiveness of RT over 60 Gy administered alone to NSCLC patients who were unfit or rejecting for combination treatment.

**Methods and materials:**

From April 2002 to July 2010, 83 patients with stage II-III NSCLC, aged over 60 years, treated by RT alone with a curative aim were analyzed. Radiation was targeted to the primary tumor and clinically involved lymph nodes. A total dose of 66 Gy in 30 fractions (2.2 Gy/fraction) was delivered once daily (5 fractions weekly). One month after completing RT, initial tumor responses were evaluated.

**Results:**

Median age of patients was 73 years (range, 60 – 82 years). The median survival time was 18.6 months (range, 2–135). The actuarial overall survival rates at 2 and 3 years were 39 % and 23 %, and cause-specific survival rate at 2 and 3 years were 57 % and 47 %, respectively. When primary tumor was controlled, the 2- and 3-year CSS were 56 % and 45 %, but 32 % and 23 % in those patients with local failure, respectively (*P* = 0.017). Additionally, the local control rate was associated with the initial tumor response (*P* = 0.01). No patient experienced grade 4+ toxicity.

**Conclusions:**

For stage II-III NSCLC patients aged over 60 years and unfit or rejecting for combination treatment, RT alone showed promising result. Long-term disease control can be expected if an early tumor response to radiation is achieved, which could result in improved overall survival rates.

## Background

Non-small cell lung cancer (NSCLC) primarily occurs in elderly patients, with a median age at diagnosis of 70 years; patients 80 years or older account for 14 % of all NSCLC patients [1, 2]. The prevalence of these cases and the resulting increased societal burden will increase as life expectancy continually improves. Patients diagnosed at advanced stages or who are medically inoperable typically receive chemotherapy and radiation therapy (RT), as randomized prospective studies have demonstrated a survival advantage for the combination of these modalities [3–5]. Despite the clear benefit, elderly patients frequently have been undertreated or do not receive chemotherapy [2]. One explanation is the poor performance status of elderly patients, which leads to intolerance to cytotoxic chemotherapy. Second, elderly patients show more pronounced chemotherapy-related toxicities, such as myelosuppression or pneumonitis [6].

Long-term survival of RT alone in inoperable NSCLC is poor, estimated at around 5 %. But, most reports of RT alone therapies for advanced NSCLC are outdated, as they involve 2-dimensional treatments and relatively low radiation doses (<60 Gy) [7–10]. Our present study aimed to review treatment results following modern RT techniques. The included patients were treated by RT alone using a 3-dimensional-conformal radiation therapy (3D-CRT), high radiation doses, and involved-field approaches. And, intensity-modulated RT (IMRT), image-guided RT (IGRT) or respiratory gating during treatment were used in selected cases, to minimize toxicities.

In this study, we investigated the efficacy and toxicity of RT alone in patients aged over 60 years with stage II-III NSCLC. With this, we aimed to discuss on the clinical outcome and find ways to increase recurrence-free survival time, in elderly NSCLC patients.

## Methods

### Patients

This study was approved by the Asan Medical Center Institutional Review Board (2012–0733). We retrospectively reviewed 112 patients with stage II-III NSCLC, aged over 60 years and were treated using conventional RT alone, from April 2002 to July 2010. Among these patients, 29 were excluded in the analysis with following reasons: follow-up period of less than six months without evidence of clinical deterioration (*n* = 7); double primary cancer (*n* = 9); RT dose under 60 Gy (*n* = 13). The reasons for dose reduction or discontinuing treatment were poor general health (*n* = 6), patient refusal (*n* = 5), and unknown (*n* = 2). A total of 83 patients were analyzed. Pretreatment evaluations included chest X-ray, chest computed tomography (CT), bronchoscopy, PET-CT, brain CT or magnetic resonance (MR), bone scan, mediastinoscopy or endobronchial ultrasound (EBUS), and pulmonary function testing. The performance status in each case was assessed using the Eastern Cooperative Oncology Group (ECOG) scale.

### Radiation therapy

Radiation was only targeted to the primary tumor and clinically involved lymph nodes. A lymph node was included in the gross tumor volume (GTV) if it was greater than 1 cm on its short axis by CT analysis, had an increased standard uptake value on ^18^FDG-PET, or was biopsy-positive. Elective nodal irradiation was not performed for any of our subjects. A total dose of 66 Gy was administered in 30 fractions (2.2 Gy/fraction) and delivered once daily (five times per week) with 4 to 6 coplanar and/or non-coplanar beams. In rare instances, the fraction sizes were adjusted from 1.8 to 3 Gy, in accordance with the tumor and patient characteristics. Later in study period, four-dimensional computed tomography (CT) was used to track tumor motion, and respiratory gating was used when tumor moves more than 1 cm during treatment.

### Follow-up and definition of recurrence

One month after completing RT, the tumor response was evaluated in each patient using CT scanning based on the Response Evaluation Criteria in Solid Tumors (RECIST) criteria. Regular follow-ups were performed that included a physical examination, complete blood count, chest X-ray, and chest CT. A follow-up was performed every three months for the first two years and then every six months thereafter. Local recurrence was defined as an increase in tumor size or tumor regrowth in radiation field that was not believed to be radiation-induced pneumonitis or fibrosis. It was comprehensively determined by radiologist and radiation oncologist.

Distant metastasis was defined as a lymph node recurrence outside of the radiation field, a lung metastasis, or a metastasis to an extra-thoracic site. Radiation-related toxicities, esophagitis, and pneumonitis were recorded based on the Common Terminology Criteria for Adverse Events (CTCAE) version 4.0 (20).

### Statistical analyses

Time was calculated from the date of RT start to the event. For local- and distant control rates, time to first recurrence was considered whereas for local- and distant-recurrence free survival, both time to first failure or death were considered as events. Survival rates were estimated using the Kaplan–Meier method. Univariate and multivariate analyses to assess predictors of survival were performed using a log-rank test and the Cox proportional hazard model. All statistical analyses were performed using SPSS version 21 software (SPSS Inc, Chicago, IL).

## Results

### Study population

The baseline characteristics of the study patients are listed in Table 1. Median age of patients was 73 years (range, 60 – 82 years), with 60 % of patients aged over 75 years. Sixty-six patients (80 %) had a history of smoking. Seventy-one patients (86 %) had stage III disease, and 17 had tumor larger than 7 cm in size. Fourty-eight percent of patients had ECOG PS 2 or 3. The most common reason for omission for the chemotherapy was poor performance status (*n* = 37). Other reasons included poor lung function (*n* = 33), cardiac disease (*n* = 7), patent’s refusal (*n* = 5), and cerebrovascular disease (*n* = 1).

**Table 1:** Patient characteristics

Characteristics	No. of patients (%)
Age, median	73 (range, 60 – 84)
<75	50 (60)
≥75	33 (40)
Sex	
Male	75 (90)
Female	8 (10)
ECOG PS	
0–1	43 (52)
2–3	40 (48)
Lung function	
Mean FEV1 (L)	1.75
Mean FEV1 (%)	72
Mean DLCO (%)	76
Smoking history	
Presence	66 (80)
Absence	17 (20)
Histology	
Squamous cell carcinoma	62 (75)
Adenocarcinoma	13 (16)
NOS	8 (9)
AJCC/UICC staging grouping	
II	12 (14)
III	71 (86)
T stage	
T1–2	33 (40)
T3–4	50 (60)
N stage	
N0–1	27 (33)
N2–3	56 (67)
Tumor size, mean (cm)	5.0 (1.5 – 12.5)
<7 cm	66 (80)
≥7 cm	17 (20)
Reasons for RT alone	
Poor performance	37 (45)
Poor lung function	33 (40)
Cardiac disease	7 (8)
Patient refusal	5 (6)
Cerebrovascular disease	1 (1)

*Abbreviations: ECOG PS* Eastern Cooperative Oncology Group performance status, *FEV1* forced expiratory volume in one second, *DLCO* diffusing capacity of carbon monoxide, *NOS* not otherwise specified, *AJCC* American Joint Committee on Cancer, *UICC* International Union Against Cancer

### Treatment and initial tumor response

Among the 83 patients in our study cohort, 77 (93 %) patients were treated using a 66 Gy at 2.2 Gy/fraction schedule, among whom 75 patients completed the course of treatment. The treatment completion rate was 97 % and the other 2 patients received 61.6 Gy. Other patients received 60 Gy at 3 Gy/fraction (*n* = 4), 65.6 Gy at 2.1 Gy/fraction (*n* = 1), or 70.4 Gy at 2.2 Gy/fraction (*n* = 1). Intensity-modulated RT was used in 3 (4 %) patients. After one month, the overall response rate was 59 %. Based on RECIST criteria, complete response (CR), partial response (PR), stable disease (SD), and progressive disease (PD) were observed in 4 (5 %), 45 (54 %), 31 (37 %), and 3 (4 %) patients, respectively.

### Survival outcomes and patterns of treatment failure

**Fig. 1:**
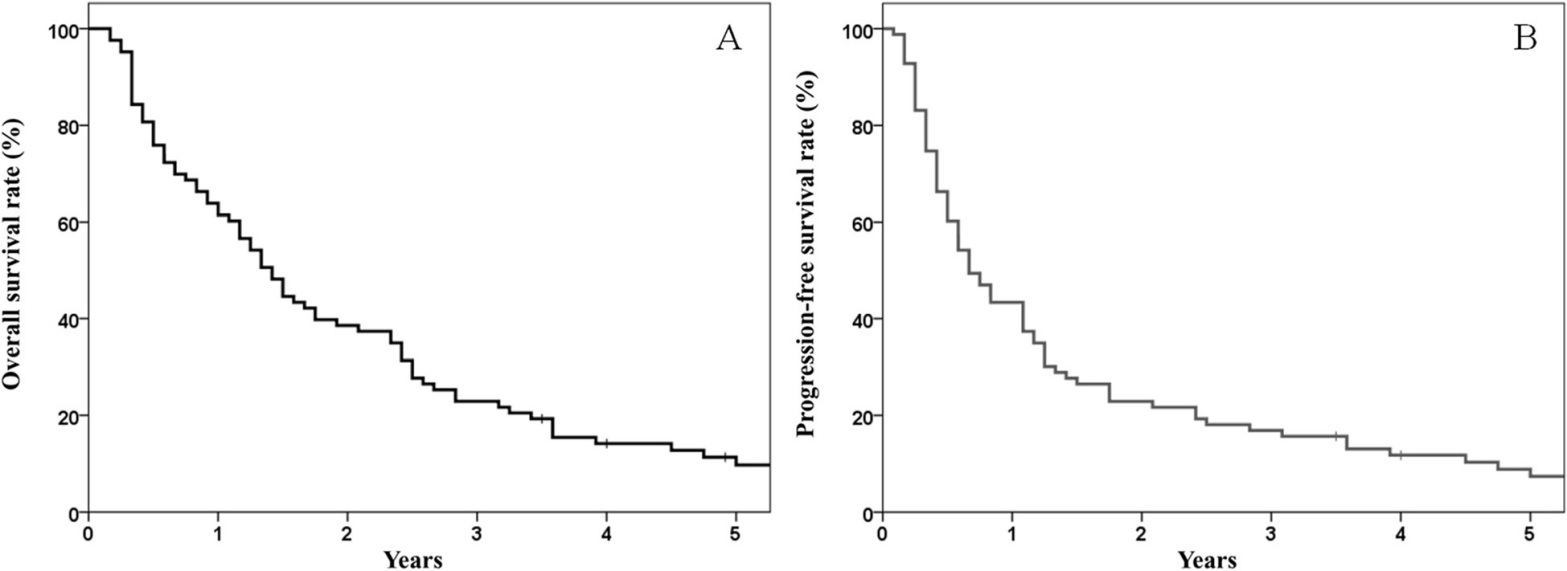
**a** Overall survival outcomes in the study patients. At 2 and 3 years these were estimated at 39 % and 23 %, respectively. **b** Progression-free survival outcomes in the study patients. The rates at 2 and 3 years were 23 % and 17 %, respectively

The median follow-up period was 70 months (range, 42 – 135) for surviving patients and 17 months (range, 2 – 135) for the whole group. The median survival time was 18.6 months (range, 2–135) for all patients. For stage II and III patients, median survival time was 24.0 months and 18.3 months, respectively. The actuarial overall survival (OS) rates at 2 and 3 years were 39 % and 23 %, respectively (Fig. 1a). The cause of death was intercurrent disease in 13 patients. The cause-specific survival (CSS) rate at 2 and 3 years were 57 % and 47 %, respectively. First recurrences occurred locally in 24 (29 %) patients, distantly in 18 (22 %) patients, and simultaneously in 5 (6 %) patients. The 2- and 3-year local control rate was 57 %, and local recurrence-free survival rates were 29 % and 18 %, respectively. The progression-free survival (PFS) rates at 2 and 3 years were 23 % and 17 %, respectively (Fig. 1b).

**Fig. 2:**
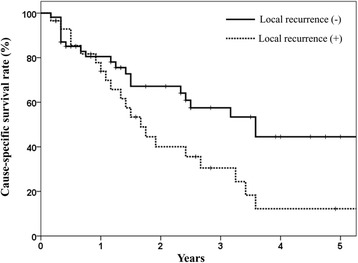
Cause-specific survivals in accordance with local tumor control. The 2- and 3-year cause-specific survival rates were 56 % and 45 %, respectively, in patients in whom the primary tumor was controlled, and 32 % and 23 %, respectively, in those patients with local failure (*P* = 0.02)

**Fig. 3:**
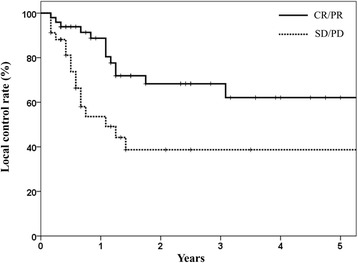
Local control rates in accordance with the initial tumor response. The local control rates at 3 years in the complete response/partial response group and stable disease/progressive disease patients were 68 % and 39 %, respectively (*P* = 0.01)

Local control was significantly associated to lung cancer-specific survival. When primary tumor was controlled, the 2- and 3-year CSS were 56 % and 45 %, but 32 % and 23 % in those patients with local failure, respectively (*P* = 0.02; Fig. 2). The corresponding values for OS was 43 %, 26 % and 31 %, 17 % (*P* = 0.45). Possibly due to small patient number, the local controllability failed to show statistically significant difference in OS in this study. Initial tumor response after completing RT was associated with local control. The local control rates at 3 years in the CR/PR and SD/PD patients were 68 % and 39 %, respectively (*P* = 0.01; Fig. 3). High local control rate of good responders resulted in trends toward better PFS. The PFS at 2 years in the CR/PR and SD/PD patients were 29 % and 15 %, respectively (*P* = 0.11). The 2- and 3-year distant metastasis-free survival rates were 33 % and 22 %, respectively. Sites of metastases included the lung (*n* = 9), liver (*n* = 5), brain (*n* = 3), bone (*n* = 3), adrenal gland (*n* = 1), multiple sites (*n* = 2). Salvage therapies for distant metastasis included chemotherapy in 11 patients, RT in 7 patients, and best supportive care in 5. Eleven patients whom salvage chemotherapy has applied to did initially not receive combination treatment, because of poor performance status or underlying disease (*n* = 7), poor lung function (*n* = 2) or patient’s refusal (*n* = 2). With caution, palliative chemotherapy with single or double regimen could be administered.

For prognostic factor analysis, age (<75 years vs. ≥ 75 years), sex, ECOG performance status, FEV1(%), pathology, tumor size (<7 cm vs. ≥ 7 cm), T- and N-stage, and initial response (CR/PR vs. SD/PD) were examined. Univariate and multivariate analysis of prognostic factors are summarized in Tables 2 and 3, respectively. Initial response after RT was the only significant factor for both PFS and CSS on multivariate analysis. None of the factors examined had difference in OS, but, good responders after RT showed trends toward better OS (hazard ratio: 1.68, confidence interval: 0.96-2.94, *P* = 0.07).

**Table 2:** Univariate analysis of prognostic factors that influence PFS, CSS, and OS

Characteristics		PFS		CSS		OS	
	N	2-year rate	*P*-value	2-year rate	*P*-value	2-year rate	*P*-value
Age			0.62		0.43		0.58
<75	50	20		54		40	
≥75	33	27		61		36	
Sex			0.82		0.49		0.86
M	75	23		43		39	
F	8	25		64		38	
ECOG PS			0.53		0.28		0.20
0-1	43	23		59		44	
2-3	40	23		53		33	
^a^FEV1(%)			0.84		0.96		0.69
≥70	45	22		50		47	
<70	33	24		46		40	
Pathology			0.81		0.92		0.71
Squamous cell carcinoma	62	24		60		63	
Adenocarcinoma	13	23		35		23	
Others	8	13		60		38	
Tumor size			0.32		0.9		0.37
<7 c m	66	18		57		36	
≥7 c m	17	41		55		47	
T-stage			0.14		0.13		0.2
1-2	33	27		63		39	
3-4	50	20		53		38	
N-stage			0.55		0.67		0.55
0-1	27	30		64		44	
2-3	56	20		53		36	
Initial response			0.11		0.07		0.17
CR or PR	49	29		71		49	
SD or PD	34	15		35		24	

*Abbreviations: PFS* progession-free survival, *CSS* cause-specific survival, *OS* overall survival, *ECOG PS* Eastern Cooperative Oncology Group performance status, *FEV1* forced expiratory volume in one second, *CR* complete response, *PR* partial response, *SD* stable disease, *PD* progressive disease

^a^FEV1 test was not performed in 5 patients

**Table 3:** Multivariate analysis of prognostic factors that influence PFS, CSS, and OS

	PFS		CSS		OS	
Characteristics	HR (95 % CI)	*P*-value	HR (95 % CI)	*P*-value	HR (95 % CI)	*P*-value
Age		0.42		0.32		0.96
<75	1		1		1	
≥75	0.82 (0.51-1.33)		0.70 (0.34-1.42)		1.01 (0.62-1.66)	
Sex		0.66		0.81		0.86
M	1		1		1	
F	0.83 (0.35-1.94)		0.86 (0.24-3.08)		1.08 (0.45-2.58)	
ECOG PS		0.27		0.14		0.90
0-1	1		1		1	
2-3	1.33 (0.80-2.19)		1.66 (0.84-3.27)		1.56 (0.93-2.60)	
^a^FEV1(%)		0.63		0.96		0.42
≥70	1		1		1	
<70	0.88 (0.54-1.45)		1.02 (0.52-2.00)		0.81 (0.49-1.34)	
Pathology						
Squamous cell carcinoma	1		1		1	
Adenocarcinoma	1.25 (0.56-2.79)	0.59	1.63 (0.62-4.29)	0.32	1.32 (0.60-2.91)	0.49
Others	0.93 (0.35-2.48)	0.88	0.70 (0.17-2.92)	0.63	0.78 (0.28-2.16)	0.64
Tumor size		0.11		0.50		0.20
<7 c m	1		1		1	
≥7 c m	0.60 (0.32-1.11)		0.75 (0.33-1.72)		0.65 (0.34-1.25)	
T-stage		0.02		0.12		0.08
1-2	1		1		1	
3-4	2.00 (1.11-3.61)		1.90 (0.85-4.26)		1.67 (0.93-2.98)	
N-stage		0.18		0.30		0.29
0-1	1		1		1	
2-3	1.42 (0.85-2.39)		1.46 (0.72-2.97)		1.33 (0.79-2.26)	
Initial response		0.02		0.02		0.07
CR or PR	1		1		1	
SD or PD	2.03 (1.15-3.58)		2.40 (1.16-4.98)		1.68 (0.96-2.94)	

*Abbreviations: PFS* progession-free survival, *CSS* cause-specific survival, *OS* overall survival, *HR* hazard ratio, *95 % CI* 95 % confidence interval, *ECOG PS* Eastern Cooperative Oncology Group performance status, *FEV1* forced expiratory volume in one second, *CR* complete response, *PR* partial response, *SD* stable disease, *PD* progressive disease

^a^FEV1 test was not performed in 5 patients

### Treatment-related toxicities

None of the patients analyzed experienced greater than grade 3 esophagitis. Grade 2 esophagitis was observed in 49 (59 %) cases. Grade 3 pneumonitis occurred in 2 (2 %) patients, and these symptoms were controllable. Grade 2 pneumonitis was observed in 11 (13 %) patients. Acute RT complications were more prominent among the older age group (≥75 years) than among the younger patients (<75 years), but the difference was not significant. Symptomatic esophagitis rate was 58 % vs. 60 % (*P* = 0.81), and acute RT pneumonitis rate was 10 % vs. 24 % (*P* = 0.08). Grade 3 pneumonitis (*n* = 2) was observed in patients older than 75 years.

## Discussion

The incidence of NSCLC in elderly patients is increasing worldwide, and the proportion of very elderly patients (80 years old or older) is also increasing [2]. Thus, there has been a growing interest in treatment of NSCLC, for patients who cannot tolerate chemotherapy. Radiation remains an attractive option either alone or combined with chemotherapy for those patients in whom a medical condition precludes surgery. The necessity for prospective trials designed for elderly is controversial. Some authors argue that elderly patients do as well as their younger counterparts, and cite retrospective data as evidence. The North Central Cancer Treatment Group (NCCTG) performed a retrospective study on 246 patients to examine the relationship between patient age and clinical outcomes. They found that elderly (≥70 years) patients had survival rates that were equivalent to those of younger individuals, except for higher hematological toxicity and pneumonitis rates (81 % vs. 62 %, *P* = 0.01). Hence, it has been argued that fit, elderly patients with locally advanced NSCLC should receive a combined-modality therapy with cautious and judicious monitoring [6, 11]. Growing evidence supports that aggressive treatment will have benefit in survival, even in elderly NSCLC patients [11–14]. But, it is obvious that elderly patients are generally not treated using a combined-modality therapy. In population-based studies of elderly patients with locally advanced NSCLC, only 30–45 % of all treated patients were subjected to chemotherapy [15, 16]. Thus, attempts to evaluate treatment outcomes for patients with locally advanced NSCLC treated using RT alone are necessary. Median age of the patients included in current study is 73 years, 60 % of patients are 75 years or older, and all of the patients were over 60 years old. In addition, half of the patients were unsuitable for chemotherapy because of underlying medical condition, as well as their poor performance status. Therefore, by reviewing the treatment results of our study cohort, we can predict treatment outcomes of RT alone in NSCLC for elderly patients.

In this study, we present survival outcomes in stage II-III NSCLC patients who were treated with RT alone using modern techniques. Treatment effectiveness of radical RT in stage I/II (T1-2/N0-1 or T3N0) medically inoperable NSCLC were summarized by Rowell et al., in Cochrane database systematic review. Two-year overall survival was 22-72 %, and median survival was 15–33 months. For patients with poor performance status, the median survival was 6–13 months. This result is poorly applied to modern treatment era, as total dose was low (≥40 Gy) and mediastinal irradiation was used. [17]. Previously reported survival outcome of stage III NSCLC patients who were treated by RT alone was relatively poor, despite high irradiated dose. Sigel et al. analyzed treatment outcome of stage III NSCLC patients using the Surveillance, Epidemiology and End Results (SEER) registry, treated between 1992 and 2007. The median OS time was 9.0 months. The survival was better when using high complexity RT, but survival time was not presented separately [18]. In phase III intergroup study by Radiation Therapy Oncology Group, Eastern Cooperative Oncology Group, and Southwest Oncology Group, median OS of stage III NSCLC patients treated by lone RT was 11.4 months for standard RT arm (60 Gy) and 12 months for hyperfractionated RT arm (69 Gy) [19]. In our present study, the median survival time was 18.6 months for all patients and 18.3 months for the 71 patients with stage III NSCLC. For stage III patients, 2-year survival rate and CSS rate was 37 % and 54 %, respectively. Despite the high proportion of patients with poor performance status (48 % of patients had ECOG ≥ 2), and old age (67 % of patients were aged over 70 years), the survival outcome was not inferior compared to previous reports of advanced NSCLC patients. This suggests that advances in RT techniques and supportive care might contribute to improved survival.

Some studies have argued that patients over 70–75 years might have benefit in survival by radiation dose over 40–50 Gy, which is considered inadequate in normal definitive treatment. But, these results were achieved mostly before modern RT technique era [20]. In a population-based study of elderly NSCLC patients, improved survival rates were only shown when high complexity RT approaches were used, and not for low complexity RT [18]. A lot of reports have shown that definitive RT dose over 60 Gy can be tolerated in older patients, as well as younger patients, and our present study result also confirms it. During study period, all patients were treated with involved-field RT without elective nodal irradiation. To reduce esophageal complication, IGRT and IMRT were used in selected cases. Respiratory gating during treatment time for minimal lung irradiation was also used when indicated. With this approach, mild esophagitis during RT were complained in 59 % of patients, but no one required parenteral nutritional support, tube feeding, or hospitalization. Symptomatic radiation pneumonitis was reported in 17 %, with 2 Grade 3 events. No grade 4+ toxicity was reported. After careful evaluation of any concurrent illness in elderly patients, definitive dose radiation can be administered with caution. In comorbid or poor performance status patients, total dose reduction or fractional dose reduction might be considered. Image-guided RT, intensity-modulated RT, or respiratory gating will be helpful in this regard. Stereotactic body radiation therapy (SBRT) might also be a suitable treatment option for early stage NSCLC. Stage IIA patients included in this study had T1N1 or T2N1 disease, thus were not indicated for SBRT. Treatment results using SBRT during same period in our institution can be found in previously published articles [21].

At the time of death, 27 of our patients exhibited evidence of local recurrence. The patients in our series who were successfully locally controlled showed improved lung cancer-specific survival. It is evident that the survival of patients with NSCLC depends heavily on local/regional tumor control. Recently, a retrospective analysis of seven Radiation Therapy Oncology Group (RTOG) prospective trials was reported. For advanced NSCLC patients who were treated using chemo-radiotherapy, there was a significant association reported between loco-regional control and survival (hazard ratio, 1.43; 95 % confidence interval, 1.28–1.61; *P* < 0.0001) [22]. The importance of local control on overall survival can also be observed from previous RT alone data. An improved 5-year CSS was reported in patients in whom the primary tumor was controlled (46 % vs. 12 %, *P* < 0.03) [23]. Additionally, several retrospective results revealed trends for increased local control followed by increased survival [24–26]. The survival advantage in higher radiation dose was also confirmed in elderly patients. Lonardi et al. treated NSCLC patients aged over 75 years, and found one-year survival rate of 28 % vs. 4 %, in patients given at least 50 Gy vs. in those treated with lower doses [20]. Krol et al. reported that out of 108 medically inoperable patients with NSCLC who were treated with RT alone, 50 (52 %) cases with a CR achieved 5 years of local relapse-free survival [27]. Among the patients in CR in that study, only two had a regional recurrence as the only site of relapse. Furthermore, Zhang et al. have reported that patients achieving CR after RT had a high OS rate and local control [10]. These results indicate that long-term survival is associated with both local control and a robust initial tumor response. Thus, further attempts including radiosensitizers and dose escalation are necessary to improve RT response for patients who are unfit for chemotherapy.

Our present study has several limitations associated with its retrospective design. This study could not prove a survival benefit of RT compared to other treatment modalities. Given the fact that included patients were not suitable for chemotherapy, the primary objective of this work was to report the treatment outcomes after RT alone in locally-advanced NSCLC patients who are unfit for chemotherapy. Compared to previous studies, relatively large number of patients and highly homogeneous RT dose were used in this study. Also, they were treated using modern techniques: 3D-CRT or IMRT, involved-field RT, and a dose greater than 60 Gy.

## Conclusions

Administering RT alone over 60 Gy for elderly NSCLC patients showed promising results, although most of these individuals experienced non-cancer-related medical problems. Long-term disease control can be expected if an early tumor response to radiation is achieved, which could result in improved OS rates. To try to improve disease control and survival in elderly patients, prospective studies of radiation dose-escalation with RT alone can be considered in selected cases of locally advanced NSCLC.

## References

[1] Havlik RJ, Yancik R, Long S, Ries L, Edwards B (1994). The National Institute on Aging and the National Cancer Institute SEER collaborative study on comorbidity and early diagnosis of cancer in the elderly. Cancer..

[2] Owonikoko TK, Ragin CC, Belani CP, Oton AB, Gooding WE, Taioli E (2007). Lung cancer in elderly patients: an analysis of the surveillance, epidemiology, and end results database. J Clin Oncol..

[3] Chemotherapy in non-small cell lung cancer: a meta-analysis using updated data on individual patients from 52 randomised clinical trials. Non-small Cell Lung Cancer Collaborative Group. BMJ. 1995;311:899–909. PMC2550915 7580546

[4] Dillman RO, Herndon J, Seagren SL, Eaton WL, Green MR (1996). Improved survival in stage III non-small-cell lung cancer: seven-year follow-up of cancer and leukemia group B (CALGB) 8433 trial. J Natl Cancer Inst..

[5] Komaki R, Scott CB, Sause WT, Johnson DH, Taylor SG, Lee JS (1997). Induction cisplatin/vinblastine and irradiation vs. irradiation in unresectable squamous cell lung cancer: failure patterns by cell type in RTOG 88-08/ECOG 4588. Radiation Therapy Oncology Group. Eastern Cooperative Oncology Group. Int J Radiat Oncol Biol Phys.

[6] Schild SE, Stella PJ, Geyer SM, Bonner JA, McGinnis WL, Mailliard JA (2003). The outcome of combined-modality therapy for stage III non-small-cell lung cancer in the elderly. J Clin Oncol..

[7] Cheung PCF, Mackillop WJ, Dixon P, Brundage MD, Youssef YM, Zhou S (2000). Involved-field radiotherapy alone for early-stage non–small-cell lung cancer. Int J Radiat Oncol Biol Phys.

[8] Dosoretz DE, Galmarini D, Rubenstein JH, Katin MJ, Blitzer PH, Salenius SA (1993). Local control in medically inoperable lung cancer: an analysis of its importance in outcome and factors determining the probability of tumor eradication. Int J Radiat Oncol Biol Phys..

[9] Talton BM, Constable WC, Kersh CR (1990). Curative radiotherapy in non-small cell carcinoma of the lung. Int J Radiat Oncol Biol Phys..

[10] Zhang HX, Yin WB, Zhang LJ, Yang ZY, Zhang ZX, Wang M (1989). Curative radiotherapy of early operable non-small cell lung cancer. Radiother Oncol..

[11] Atagi S, Kawahara M, Yokoyama A, Okamoto H, Yamamoto N, Ohe Y (2012). Thoracic radiotherapy with or without daily low-dose carboplatin in elderly patients with non-small-cell lung cancer: a randomised, controlled, phase 3 trial by the Japan Clinical Oncology Group (JCOG0301). Lancet Oncol..

[12] Domingues PM, Zylberberg R, da Matta de Castro T, Baldotto CS, de Lima Araujo LH (2013). Survival data in elderly patients with locally advanced non-small cell lung cancer. Med Oncol..

[13] Strom HH, Bremnes RM, Sundstrom SH, Helbekkmo N, Aasebo U (2015). How Do Elderly Poor Prognosis Patients Tolerate Palliative Concurrent Chemoradiotherapy for Locally Advanced Non-Small-Cell Lung Cancer Stage III? A Subset Analysis From a Clinical Phase III Trial. Clin Lung Cancer..

[14] Kang KM, Jeong BK, Ha IB, Chai GY, Lee GW, Kim HG (2012). Concurrent chemoradiotherapy for elderly patients with stage III non-small cell lung cancer. Radiat Oncol J..

[15] Davidoff AJ, Gardner JF, Seal B, Edelman MJ (2011). Population-based estimates of survival benefit associated with combined modality therapy in elderly patients with locally advanced non-small cell lung cancer. J Thorac Oncol..

[16] Ramsey SD, Howlader N, Etzioni RD, Donato B (2004). Chemotherapy use, outcomes, and costs for older persons with advanced non-small-cell lung cancer: evidence from surveillance, epidemiology and end results-Medicare. J Clin Oncol..

[17] Rowell NP, Williams CJ (2001). Radical radiotherapy for stage I/II non-small cell lung cancer in patients not sufficiently fit for or declining surgery (medically inoperable): a systematic review. Thorax..

[18] Sigel K, Lurslurchachai L, Bonomi M, Mhango G, Bergamo C, Kale M (2013). Effectiveness of radiation therapy alone for elderly patients with unresected stage III non-small cell lung cancer. Lung Cancer..

[19] Sause W, Kolesar P, Taylor SI, Johnson D, Livingston R, Komaki R (2000). Final results of phase III trial in regionally advanced unresectable non-small cell lung cancer: Radiation Therapy Oncology Group, Eastern Cooperative Oncology Group, and Southwest Oncology Group. Chest..

[20] Lonardi F, Coeli M, Pavanato G, Adami F, Gioga G, Campostrini F (2000). Radiotherapy for non-small cell lung cancer in patients aged 75 and over: safety, effectiveness and possible impact on survival. Lung Cancer..

[21] Song SY, Choi W, Shin SS, Lee SW, Ahn SD, Kim JH (2009). Fractionated stereotactic body radiation therapy for medically inoperable stage I lung cancer adjacent to central large bronchus. Lung Cancer..

[22] Machtay M, Paulus R, Moughan J, Komaki R, Bradley JE, Choy H (2012). Defining local-regional control and its importance in locally advanced non-small cell lung carcinoma. J Thorac Oncol..

[23] Sibley GS, Jamieson TA, Marks LB, Anscher MS, Prosnitz LR (1998). Radiotherapy alone for medically inoperable stage I non-small-cell lung cancer: the Duke experience. Int J Radiat Oncol Biol Phys..

[24] Hayakawa K, Mitsuhashi N, Saito Y, Nakayama Y, Furuta M, Sakurai H (1999). Limited field irradiation for medically inoperable patients with peripheral stage I non-small cell lung cancer. Lung Cancer..

[25] Kaskowitz L, Graham MV, Emami B, Halverson KJ, Rush C (1993). Radiation therapy alone for stage I non-small cell lung cancer. Int J Radiat Oncol Biol Phys..

[26] Qiao X, Tullgren O, Lax I, Sirzen F, Lewensohn R (2003). The role of radiotherapy in treatment of stage I non-small cell lung cancer. Lung Cancer..

[27] Krol AD, Aussems P, Noordijk EM, Hermans J, Leer JW (1996). Local irradiation alone for peripheral stage I lung cancer: could we omit the elective regional nodal irradiation?. Int J Radiat Oncol Biol Phys..

